# Seed Structural Variability and Germination Capacity in *Passiflora edulis* Sims f. *edulis*

**DOI:** 10.3389/fpls.2020.00498

**Published:** 2020-05-29

**Authors:** Nohra Rodríguez Castillo, Luz Marina Melgarejo, Matthew Wohlgemuth Blair

**Affiliations:** ^1^Laboratory of Plant Physiology and Biochemistry, Department of Biology, Universidad Nacional de Colombia, Bogotá, Colombia; ^2^Department of Agricultural and Environmental Sciences, Tennessee State University, Nashville, TN, United States

**Keywords:** morphometric variability, seed anatomy, germination, viability, descriptors

## Abstract

Purple passion fruit, *Passiflora edulis* Sims f. *edulis*, is an important semi-perennial, fruit bearing vine originating in South America that produces a commercial tropical juice pulp for international and national consumption. Within the round purple passion fruit are a large number of membranous seed sacs each containing individual seeds. Little is known about the seed anatomy of the commercial passion fruit, differences between wild collected and commercial types, and its effect on seedling germination. Therefore, our main objective for this study was to analyze the seed anatomy variability of different germplasm as well as the effect on viability and germination of the seeds of this species. Germplasm was evaluated from three sources: (1) commercial cultivars grown in current production areas, (2) genebank accession from the national seed bank, and (3) landraces collected across different high and mid-elevation sites of the Andean region. A total of 12 morphometric descriptors related to seed anatomy were evaluated on the 56 genotypes, of which three were most informative: Angle to the vertex which is related to the shape of the seed, the thickness of the tegument and the horizontal length; separating the seed according to its source of origin. Germination was found to be positively correlated with the number (*r* = 0.789) and depth (*r* = 0.854) of seed pitting. Seeds of the commercial cultivars had more seed pits and higher germination compared to seeds of landraces or genebank accessions showing a possible effect of domestication on the crop. Interestingly, passion fruits often germinate during the rainy season as escaped or wild seedlings especially in the disturbed landscapes of coffee plantations, so some dormancy is needed but faster germination is needed for intensive cultivation. Harnessing passion fruit diversity would be useful as the semi-domesticated landraces have valuable adaptation characteristics to combine with rapid germination selected in the commercial cultivars. The variability of seed pitting with cultivars more pitted than landraces possibly resulting in faster germination may indicate that purple passion fruit is still undergoing a process of selection and domestication for this trait.

## Introduction

Seed structural variability is related to germination capacity and can be a step in the process of domestication that distinguishes wild accessions from semi or fully domesticated crops (Koomneef et al., 2002). Fruit crops propagated by seed may be subject to this type of selection given that they are often grown in hedgerows rather than in row crop agriculture. Purple passion fruit, *Passiflora edulis* Sims f. *edulis*, is an important seed-propagated, semi-perennial fruit bearing vine, which bears fleshy, and spherical “pepo type” berries with purple rinds that are filled with up to 200 small black seeds (Morton, 1987). The species originated in South America and exports from there and other tropical regions make it a major source of fruit pulp (Thokchom and Mandal, 2017). While passion fruit species can be propagated clonally (Salomão et al., 2002; Gelape et al., 2019), to date there are no seedless varieties and all are seed propagated or grafted onto seedlings. Thus, seed physiology is important to study.

Within the purple passion fruit are the many membranous seed sacs containing individual seeds (Morton, 1987). These are produced within a single ovary after self-pollination assisted by carpenter bees (Garcia, 2010; de Lira et al., 2016). The amount of seeds per fruit and the seed size and shape typically vary within and between species of *Passiflora* (Pérez-Cortéz et al., 2002). When pressed for juice the pithy rind is cracked and liquid is removed from the sacs with the seed remaining as a by-product (Macedo et al., 2015). Although the seeds are edible they are poorly studied but contain interesting nutritional and biochemical properties (Devi et al., 2018). The species is of great importance for varietal maintenance and production (Morton, 1987) but has spatula type embryos that are inhibited from germination by physical and physiological dormancy (Black, 1972; Baskin and Baskin, 1998; Koomneef et al., 2002).

Little is known about the seed anatomy of purple passion fruit and its effect on seedling germination or its variability among landraces and wild collected accessions. Seed characteristics are key to the seed dormancy, seedling propagation and plant survival of passion fruits (de Oliveira et al., 2010), and plants in general (Baskin and Baskin, 1998). The sweet flesh around the seeds of passion fruits are important for seed dispersal by birds, serving as an attractant and motivator for them to pick through the thick egg-like rind around the fruit (Torres, 2018). The round fruits can fall off naturally from the vine after a long period of drying on the plant and can roll downhill to settle in a moist ditch, where they are likely to germinate as a mass of seedlings (Veiga et al., 2013). This form of whole fruit dispersal can be considered advantageous in the natural environment of *Passiflora* spp., which grow mostly in hillside cloud forests of South America (Delanoy et al., 2006).

Dormancy and germination rates are other factors that can control the number of progenies a plant can make and is variable both in space and time with large environment effects (Smẏkal et al., 2014). The semi-domesticated passion fruit have been shown to have strong dormancy effects (de Oliveira et al., 2010; Torres, 2018). Slow germination for wild passion fruit seed can be overcome by stratification and temperature (Ramírez et al., 2015). The effect of seed structure on dormancy is less well studied than biochemical factors that inhibit germination in cultivated passion fruit (Araújo et al., 2007). Seed coat shape and thickness have been shown in some other plant families to affect germination (Chernoff et al., 1992; Gabr, 2014). In this study, we observed differences in the seed coat pitting abundance of the lignified tegmen of purple passion fruit genotypes which relate to viability and ultimately to germination. Seed coat characteristics of dry seed influence electrical conductivity and desiccation tolerance, which in turn affect seed preservation strategies as well as viability/germination (Baskin and Baskin, 1998; Veiga et al., 2013; Mira et al., 2015).

Dry seed characteristics, the focus of this study while important for plant establishment can be related to fruit characteristics as well (Macedo et al., 2015). The ratio of seed to pulp and juice has been investigated in purple passion fruit by comparing fresh mucilaginous and dry seed weight and is one factor that distinguishes commercial cultivars and landraces, and economic yield of the crop (Rodríguez et al., 2019, 2020). Seed/pulp ratio along with fruit size and fruit wall thickness are important parameters for crop improvement in breeding programs in East Africa and Asia (Matheri et al., 2016; Liu et al., 2017) but little work has been done on seed size, germination rate and their relation to pulp mass.

The objective of this research was to evaluate purple passion fruit genotypes from different sources for seed coat morphology and other seed characteristics, and associate these with seed viability as reflected in dormancy and time to or capacity to germinate. The germplasm sources used were commercial cultivars, genebank accessions and landraces collected from around Colombia. The hypothesis of lower dormancy and faster germination for commercial cultivars of purple passion fruit seeds was tested by comparing the average for these genotypes compared to the other two groups. The seed was not pre-treated and therefore differences may be a result of selection during commercialization or even during domestication of purple passion fruit genotypes. While seed morphology has been studied as a characteristic to distinguish different species within the Passifloraceae family, this is the first study to evaluate intra-specific variability for seed morpho-anatomical traits and adds to the data for tropical plants found in Baskin and Baskin (1998). This study showed that it was important to understand the processes of seed trait selection in cultivar development and to conserve the purple passion fruit landraces and genetics resources in the Andean region both for conservations and production of the crop. In Colombia, the purple passion fruit is the fourth most important export in the category of fresh or processed fruits, after bananas, berries, and pineapples (Fischer et al., 2009). Despite its importance, the purple passion fruit remains poorly investigated agronomically as well especially compared to the yellow passion fruit, which is more prevalent throughout tropical lowland areas of Brazil, Central America, and the Caribbean (Cerqueira et al., 2014; Marostega et al., 2017). Although purple passion fruit is now found in the highland tropics of Africa and sub-tropical regions, its original range was in mid to higher altitude elevations of the Andes (Franco et al., 2014).

## Materials and Methods

### Plant Material and Sites of Collection

Germplasm of purple passion fruit (*P. edulis* f. *edulis* Sims) was collected from three different sources: 8 commercial cultivars from farmers’ fields in the department of Cundinamarca and Boyacá, 14 genebank accessions were from the field grown *in situ* genebank collection kept by Agrosavia (ex. Corpoica) at the Llanogrande Experiment station in Rionegro, Antioquia and 34 were landraces from all over Colombia collected in the year prior to the study. This gave a total of 56 genotypes. The landraces were collected from backyard gardens, roadsides or small farms where the purple passion fruits were semi-wild or isolated plants rather than the main crop. This was common to many departments in the country since purple passion fruit is widely distributed in both the coffee region and vegetable or potato/corn and bean farms of the highland region. Departments of Colombia represented by the landraces included Antioquia (2 genotypes), Boyacá (6), Cauca (2), Cundinamarca (8), Huila (7), Nariño (5), Norte de Santander (2), Putumayo (4), Quindío (2), Risaralda (2), Santander (1), and Tolima (1) as found in Supplementary Table 1.

### Seed Viability and Germination

Seed viability was determined for the 56 genotypes by staining with 1% tetrazolium, an accepted process according to Sawma and Mohler (2002). Percentage seed germination was evaluated on a standard medium based on the method of Suárez and Melgarejo (2010) using 100 seeds from every accession grown on experimental plots described by Rodríguez et al. (2019, 2020). We were studying dried seed with mucilage and pulp removed and therefore our focus was on the pitted seed coat/integument alone.

### Anatomical and Morphological Traits

Dry seed from the same source described above were evaluated for a total of 12 morphological traits: seven of these were morphometric descriptors suggested by Tangarife et al. (2009), Santos et al. (2014), Marostega et al. (2017); while five were novel characteristics of seed shape and seed pitting that were new to this study. Two traits were evaluated qualitatively; namely FOR = seed form and COL = seed color. Seed form was based on shapes being divided into elliptical or semi-elliptical, and closer to oval. Given the pointed ends to the passion fruit seed none were truly oval but rather semi-elliptical. Seed color were ranked as black or brown and had no arils on them. Seed pits were present on all accessions so were evaluated for their prevalence or area coverage of the seed coat and depth into the seed coat (integument). These pits consist in semi-circular indentations in the seed coat. After this, 10 traits were measured as quantitative variables and were given the following abbreviations: PCI = fresh weight of 100 seed; LOH = seed width; LOV = seed height; ASE = seed area; PSE = seed perimeter; NSU = number of seed pits per side; ASU = area of seed pits; PSU = perimeter of seed pits; GTE = integument width; and AVE = angle of seed tips. This last trait measured the seed shape as characterized by the seed angle between the two vertices formed by the seed tips. Each quantitative trait was measured on 100 seed in three replicates and averages reported for 56 genotypes.

### Statistical Analysis

The data were analyzed for descriptive statistics comparing the three groups of purple passion fruit genotypes. Quantitative data were tested for normality and significance in analyses of variance (ANOVA) along with positive or negative correlation values with R software. The quantitative trait values for each group were then compared with mean separation using Duncan tests in SAS version 9.4 (SAS Institute, Cary, NC, United States). The full set of traits was also used for Multidimensional scaling (MDS) tests done with average values to evaluate the diversity between characteristics, genotypes and germplasm groups, based on RWizard software^1^. The MDS defined each trait as a vector whose proximity to other vectors for other traits reflected associations between traits confirming correlations, and their relative importance for the phenotyping, individually or in conjunction. Variance inflation factors (VIF) were estimated to determine which traits explained the major portion of variability and subsequently a principal component analysis (PCA) was done with the same software to separate germplasm groups (cultivars, genebank accessions, and landraces) and to identify outlier genotypes. Note that seed was dried to determine seed water content comparing fresh to dry weight of 100 seed (PCIs) and expressing on a wet-weight basis moisture content in percent (%) with the formula: [(W2–W3)/(W2–W1)] × 100; considering that W1 = weight of container with lid; W2 = weight of container with lid and sample before drying; and W3 = weight of container with lid and sample after drying.

## Results

### Seed Viability and Germination Tests

Seed viability varied significantly (*p* < 0.001) based on the source of seeds tested and group of genotypes (Table 1), being highest on average for commercial cultivars (91%), followed by landraces (71%), and genebank accessions (65%). Similarly, germination rates presented highly significant differences (*p* < 0.001) between germplasm groups with 80%, 63%, and 59% average germination for commercial cultivars, landraces and genebank accessions, respectively (Table 2). Skewing, a form of non-normality in a population, was found for the distribution of genotypes’ seed viability in the genebank group but not in the landraces or cultivars. For the germination rate, skewing was much less evident. Slight negative kurtosis was found for the distribution of values for both variables.

**TABLE 1:** Sum of Squares from the Analyses of Variance (ANOVAs) for 10 seed characteristics evaluated on 56 genotypes of purple passion fruit (*Passiflora edulis* f. *edulis* Sims) in three germplasm groups made up of 8 commercial cultivars, 14 genebank accessions and 34 landraces.

**Variable**	**Df**	**ASE**	**ASU**	**LOH**	**LOV**	**NSU**	**PCI**	**PSE**	**PSU**	**Germination**	**Viability**
Within groups	55	0,00091^*ns*^	0,0013***	0,078*	0,047*	76,455***	0,163*	0,029^*ns*^	0,00037*	434,36*	166,48*
Between groups	2	0,0251*	0,011***	1,989*	1,282*	1876,8***	2,481***	0,668*	0,0071*	3187,18***	3862,06***

Abbreviations: ^*ns*^not significant, *significant (0.01 < *P* < 0.05), **high significance (0.001 < *P* < 0.01), and ***highly significant (*P* < 0.001), Df = degrees of freedom; Variables: ASE = seed area, ASU = area of seed pitting, LOH = seed length on the horizontal axis, LOV = seed width on the vertical access PCI = fresh dry weight of 100 seeds, NSU = number of seed pits, PSE = seed perimeter, and PSU = seed pit perimeters.

**TABLE 2:** Average seed viability and germination percentage based on three germplasm groups of purple passion fruit (*Passiflora edulis* f. *edulis* Sims) made up of 8 commercial cultivars, 14 genebank accessions and 34 landraces.

	**Germplasm Group**	**N**	**Mean (%)**	**Variance**	**Std. Dev.**	**Std. Error**	**CV**	**Skewing**	**Kurtosis**
Seed Viability	Cultivars	8	91.04	19.27	4.39	1.79	5.2	–0.05	–1.88
	Genebank entries	14	64.56	128.25	11.32	4.28	18.48	0.37	–1.78
	Landraces	34	71.29	247.49	15.73	2.7	21.82	–0.4	–1.32
Germination Percent	Cultivars	8	80.05	62.01	7.87	3.21	10.57	–0.5	–1.18
	Genebank entries	14	59.28	88.18	9.39	3.55	16.72	–0.19	–1.54
	Landraces	34	62.93	101.65	10.08	1.73	16.05	0.15	–1.32

**P* < 0.01, Based on *n* = 100 seed per accession.*

### Morphological Seed Variability

Seeds of purple passion fruit were found to vary in seed shape from elliptical to semi-elliptical or oval; and in seed color from either brown to black (Table 3). Determination of elliptical or oval shape was based on measurements of the vertical and horizontal cross section widths and lengths, angle between the vertices at the seed tips and other morphological characteristics. Observable differences in seed lengths, seed widths and resulting seed sizes measured as area of each genotypes’ seeds measured on one side, gave an overall picture of the morphological differences in passion fruit types. These variables were associated with each other and with the qualitative differences seen in the figure part of the Table 2. Larger elliptical black seed were the only kind of seed in commercial cultivars and genebank entries. All seed had some degree of seed pitting.

**TABLE 3:** Qualitative traits measured on the seed of 56 genotypes of purple passion fruit (*Passiflora edulis* f. *edulis* Sims).

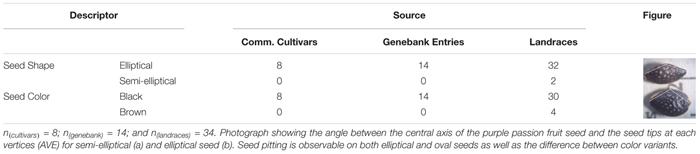	

Semi-elliptical black seeds were found in two landraces, BUN009 from Boyacá, and BUN037 from Huila (for geographic coordinates see Supplementary Table 1). Meanwhile elliptical brown seed was found for four genotypes: namely BUN002 from Cundinamarca, BUN032 from North Santander, BUN036 from Cauca, and BUN040 from Huila. All other genotypes were black seeded with elliptical seed, the only phenotype found among commercial cultivars and genebank accessions. Seed pits were common across colors.

**FIGURE 1:**
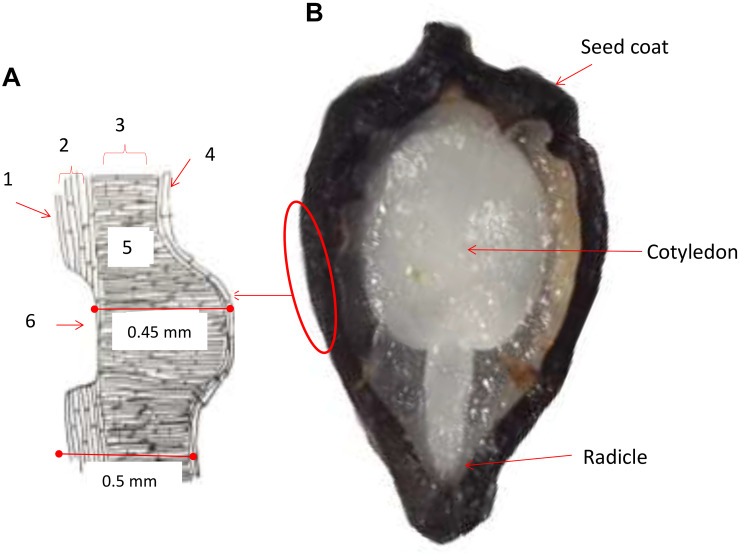
Cross sections of a purple passion fruit (*Passiflora edulis* f. *edulis* Sims) seed coat **(A)** and the entire seed **(B)**. In the diagram of the seed coat to the left, a seed pit is shown. The seed pit is the depth of the seed coat cuticle. Approximate widths of the seed coat are 0.45 mm below the seed pit and 0.5 mm on either side of the seed pit. Numbers around the diagram indicating the following: 1 = exotesta; 2 = mesotesta; 3 = exotegmen; 4 = hyaline cells; 5 = palisade cells; and 6 = seed pit. The radicle and cotyledon of the seed embryo indicated in the seed cross section.

A diagram was drawn based on typical seed structure from our histological analysis to show the seed coat and cell layers below (left part of Figure 1). The first layer consisted of an external wax coating that was continuous and highly resistant to cracking and fractures. This was subtended by an exotesta and mesotesta layer of sclereid cells, then by a layer of palisade cells presumed to have originated from the integument based on previous studies (Cárdenas-Hernández et al., 2011). The palisade cells were oriented perpendicular to the sclereid cells. Seed pits consisted of an embossed pattern of indentations found in the sclereid cell layer with fewer and more narrow cells subtended directly by the exotegmen tissue and narrow layer of hyaline cells. Seed pitting was found on both sides of the seed and was evaluated for pit number, perimeter and area. All together, the three layers making up the seed coat varied in thickness based on the pattern of pitted and non-pitted areas. Every layer presented differences in the form of the cells, color and thickness, between the sections of the basal and medium parts of the seeds, with differences in width notable between accessions. Overall, the seed coat measurements were 0.45 mm thick on average below the seed pit; while in non-pitted areas the thickness of the seed coat was 0.50 mm on average. This is thicker than what has been found in *P. ligularis*, the granadilla from Cárdenas-Hernández et al. (2011), upon which our studies were based.

### Correlation Values

During the seed viability testing, the seed thickness was found to differ and influence seed germination percentage and time to completely germinate, which fluctuated between 1.5 and 3.5 months. The seed coat also varied in amount, location and depth of seed pitting which in turn affected the seed coat thickness at the seed pits and between them. This was observable upon inspection by visible microscopy.

Highly significant (*P* < 0.001) positive correlation values were found for the germination percentage with the area covered in seed pitting (*r* = 0.854) as well as number of seed pits (*r* = 0.789, *P* < 0.001; Table 4); but not between percentage germination and seed size or length. Seed germination was also correlated significantly and positively with perimeter of seed pits (*r* = 0.432, *P* = 0.047) as well as fresh seed weight (*r* = 0.547, *P* = 0.023).

**TABLE 4:** Correlation values (above diagonal) and corresponding significance (*p*-values, below diagonal) for seed traits measured on 56 genotypes of purple passion fruit (*Passiflora edulis* f. *edulis* Sims).

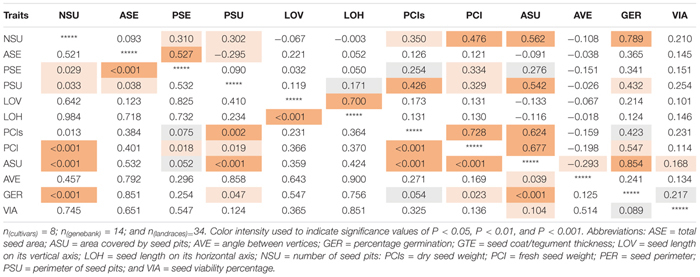

The high correlation value showed that genotypes with the most seed pitting, germinated much more quickly than those without much pitting. Microscopy showed that seed pits were the areas of thinner seed coat thickness and might capture water or allow water entry and faster seed imbibing. The seed pitted area, number of seed pits and seed pit perimeter were all positively correlated with each other as were fresh and dry seed weights and seed perimeter but not with angle between seed vertices, a measure of seed shape.

As expected, angle between vertices as a quantitative trait was related to the shape of the seed as a qualitative trait. Classification of the passion fruit genotypes into two groups found semi-elliptical seed with seed vertices at an angle ∼140° and elliptical seed with seed vertices at an angle ∼110°. Therefore, visual observation of the trait of semi versus elliptical seed was just as good as measuring angle between seed vertices.

### Principal Component Analysis Relationships

**FIGURE 2:**
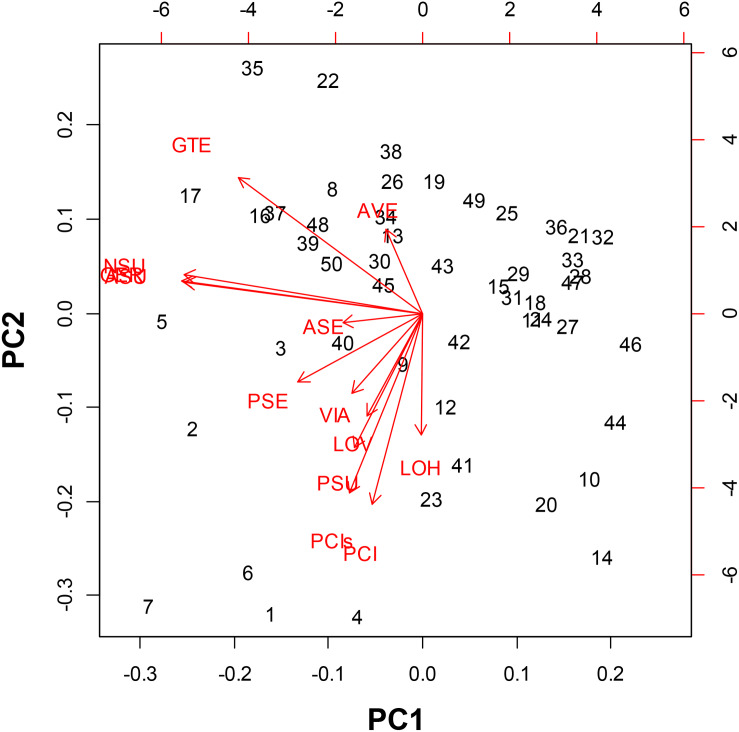
Principal Component Analysis for seed morphology traits evaluated on 56 genotypes of purple passion fruit (*Passiflora edulis* f. *edulis* Sims.), with numbers representing cultivars, landraces and accession, and based on the following characteristics: AVE = angle between seed vertices, GTE = seed coat/gross tegument width, NSU = number of seed pits per side, ASU = area of seed pits per side, GER = percentage (%) germination, PSE = perimeter of the seed, ASE = area of the seed, VIA = percentage (%) viability, LOV = length of the seed on vertical axis, LOH = length of the seed on the horizontal axis, PSU = perimeter of the seed pits, PCI = one hundred (100) fresh seed weight; and PCIs = one hundred (100) dried seed weight.

In the PCA we could distinguish the seed morphological characteristics most related to classifying the genotypes of purple passion fruit (Figure 2). Principal component 1 was explained by the angle of vertices and seed coat (tegument) thickness while principal component 2 was explained by the variables seed length seed width as well as fresh and dry seed weight.

These first two components and therefore these main variables, explained 85% of the variability found for seed phenotypes. In looking at the relationships between individual traits based on the proximity of their vectors we found relationships between the number of seed pits, the area covered by seed pits and percentage germination. These were the most important traits in our study in terms of variability.

Meanwhile, component traits of seed size were associated, such as seed length on vertical and horizontal axis as well as fresh and dry seed weight. Perimeter of the seeds and perimeter of the seed pits were associated with the primary group of traits as well and they were closely linked to seed viability.

Angle between vertices and seed coat thickness were outliers that were not associated with other traits showing that the genetic control for this trait is likely to be different than for the traits of number of seed pits and area covered by seed pitting or those genes controlling seed size. The vector for perimeter of seed pits was associated with those for vertical length and horizontal length as well as fresh and dry seed weight showing some association for seed size factors. These were also associated with area and perimeter of the seed. Epistatic interaction between traits are likely for the seed size traits as one group and the seed pitting traits as another group.

The most distinct genotypes in the PCA were BUN001, BUN002, BUN003, BUN004, BUN005, BUN006, and BUN007 in the left bottom quadrant; while BUN10, BUN12, BUN14, BUN41, BUN44, and BUN46 were distinct in the right bottom quadrant. Apart from these only BUN17, BUN22, and BUN35 were distinct toward the left upper quadrant.

Among the three germplasm groups, the commercial cultivars had significantly greater seed pitting, more seed pitted area, larger seed size, and associated longer seed perimeter traits than landraces or genebank accessions. Landraces, on the other hand, had the smallest values for all these characteristics, and genebank accessions were intermediate between landraces and commercial cultivars for the seed size and seed pitting traits. Meanwhile, no significant differences (*P* = 0.8) were found between groups for seed moisture content and fresh or dry seed weight (PCI and PCIs, respectively).

### Variance Inflation and Multi-Dimensional Scaling

**FIGURE 3:**
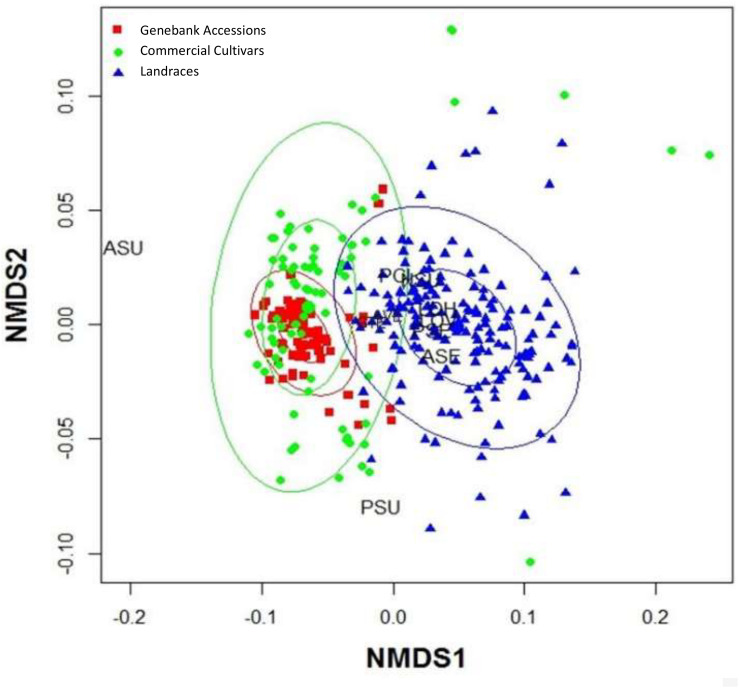
Multidimensional Scaling (MDS) analysis for the seed traits contributing highest variance to the analysis of commercial cultivars, genebank accessions and landraces of purple passion fruit, *Passiflora edulis* Sims. f. *edulis*, namely ASU = area of seed pitting, AVE = angle between seed vertices, PCI = fresh dry weight of 100 seeds, GTE = seed coat thickness, NSU = number of seed pits, LOH = length of the seed on the horizontal axis, LOV = length of the seed on the vertical access, PSE = seed perimeter, ASE = seed area, and PSU = seed pit perimeters. Number of measurements: *n*_(cultivars)_ = 80; *n*_(genebank)_ = 280; and *n*_(landraces)_ = 340.

The VIF was determined for each of the quantitative morphological variables, in order to establish which ones explained the diversity among the accessions. The most significant variables including average angle of divergence, seed coat thickness, length of the seed on the horizontal axis and seed fresh weight, were then used in a MDS analysis (Figure 3).

The advantages of an MDS were that qualitative and quantitative traits could be analyzed together by comparing and contrasting their distributions across the population of genotypes, without any preconditions or assumptions made about their relationships based on the sampled population.

### Geographical Distribution of Genotypes by Seed Size

**FIGURE 4:**
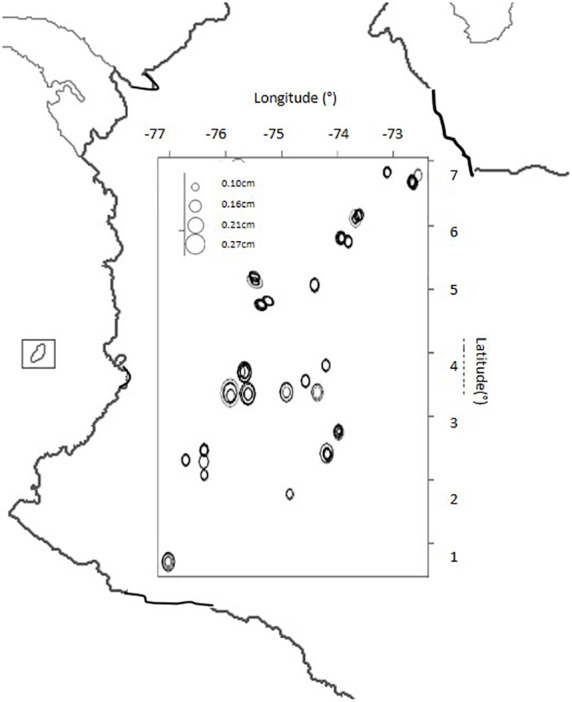
Seed size (in cm) distribution for landrace genotypes of purple passion fruit, *Passiflora edulis* Sims. *F. edulis*, collected from Colombia based on geographical collection site with latitude and longitude numbers (in degrees) given to the top and right of the boxed area.

The seed size descriptor was plotted by geographical coordinates of latitude and longitude for the collection site of each genotype on a map of Colombia (Figure 4). The main trend was that larger seeded genotypes with an area of 0.21 to 0.27 mm^2^ were distributed only in the Central Andes Mountains while smaller seeded genotypes with an area of 0.1 to 0. 16 mm^2^ were distributed throughout all other regions.

Comparing the geographical origins of the genotypes used in the study by regions shown on the map figures, we found that seeds were larger in the Central region of the Colombian Andes, probably due to the type of climate or soils in this region; and to the commercial cultivars originating in this region. Landraces from other parts of the country are more likely to be from ecologically less favorable regions outside the production zones and therefore, have unselected and smaller sized seed.

## Discussion

A major accomplishment of this study was to characterize seed of a large number of genotypes of purple passion fruit for characteristics important to establishment of seedlings and potentially domestication. Seed germination, dormancy, and viability plus subsequent seedling vigor are important limitations for many wild and semi-domesticated plant species (Baskin and Baskin, 1998). Among our observations about seed, we found that the purple passion fruit genotypes from our collection required at least 1.5 months to germinate but could take as long as 3.5 months or could remain dormant. Late and delayed germination was also found by Montaña et al. (2014), who observed that 50% of their seed germinated after 2 months. The rate gradually increases with longer time. Some seeds in our study only germinated after 4 months but were not used for evaluation. Previous studies of purple passion fruit also have observed long physiological dormancy periods prior to germination (Delanoy et al., 2006; Araújo et al., 2007; Barbosa et al., 2012; Veiga et al., 2013).

Another finding of our study was that the low germination values found in purple passion fruit were especially pronounced in landraces compared to commercial cultivars. Montaña et al. (2014) found similar slow germination for landraces with 50% of seeds germinating at 48 days after sowing. Current studies of passion flower seed germination are trying to determine the type of dormancy in *P. edulis* and its relatives. Some authors have suggested that passion fruit seeds are intermediate between rapid germination typical of fully domesticated annual crops and slow germination typical of many wild or semi-domesticate seeds (Silva et al., 2015). The intermediate nature of passion fruit seed is important for conservation efforts, however, more importantly new protocols for successful germination are needed (Meletti et al., 2007; Magalhães, 2010; Veiga et al., 2013; Moreno et al., 2015).

In support of a hypothesis of semi-recalcitrance, this study found that seed viability was slightly higher (cultivars 91%; landraces 71%, and genebank accessions 65%) than seed germination (cultivars 80%; landraces 63%, and genebank accessions 59%); suggesting that many seeds were alive but dormant. Barbosa et al. (2012) and Mira et al. (2015) reported percentage viability greater than 75% using conductivity tests, despite low germination rates of 40%. Germination capacity and seed viability are thought to vary depending on seed type among families of plants, or within species and genera (Baskin and Baskin, 1998). Ramírez et al. (2015) have suggested a role for mycorrhizae in germination of passion fruit seeds. Overall the purple passion fruit can be considered to have strong dormancy that is both physical and physiological in nature (Baskin and Baskin, 1998). The low germination values seen for some genebank accessions in our study (Average 59%) could be due to seed shipment and storage conditions but commercial and landrace types were fresh seed.

Among the groups of genotypes used in this study, we observed that the viability and germination was higher in the commercial cultivars (91 and 80%, respectively) compared to the landrace (74% and 63%) or genebank accessions (65% and 59%). This difference could be explained by selection during the domestication process; whereby commercial cultivars have been selected for higher seed germination so as to be good genotypes for crop establishment, better than landraces. Other possibilities could relate to genetic diversity among the groups, since passion fruit flowers are known to be primarily inbreeding but up to 70% outcrossing (Bruckner et al., 1995), crosses between low and high germination genotypes are needed. Seed quality can also relate to plant physiological differences during fruit production (Carvalho and Nakagawa, 2012; Lima et al., 2017; Fischer et al., 2018). Genebank accessions were briefly stored prior to the tests so some difference with landraces could be due to seed age (Larré et al., 2007).

In other observations from this study, seed size was variable between the groups of genotypes. Seed size and weight compared to fruit weight is a determinant of juice yield (Macedo et al., 2015). Juicing success is also based on fruit size and weight or percentage of the fruit made up of the fruit wall or shell. Tradeoffs in aryl versus seed production determine plant nutritional traits of seeds since these are higher in proteins and starches requiring more nitrogen and remobilized carbon, respectively, while the pulp is higher in sugar content (Devi et al., 2018). Given the characteristics of many seed per fruit and sweet aryls surrounding each seed, evolutionary forces would tend to increase the number of seeds for the dispersal by birds but decrease the seeds for the germination from fallen and rotting fruits. Bird dispersal could also scarify seed through the digestive tract making seed coat thickness and dormancy important issues in the evolution of passion fruit species. In contrast, seedlings germinating from fallen fruit would rely on natural weathering for seed germination. The inter-relationship of all these factors on landraces, which often grow naturally in coffee plantations, would be expected to be higher than on commercial cultivars where farmers select seed harvested from fruits dried and processed in artificial settings, separate from natural processes. These evolutionary forces among others (Baskin and Baskin, 1998) could explain the variations in seed anatomy and morphology which were observed.

Passion fruit species are known to be in the process of diversification (Bruckner et al., 1995) and evolution (Cerqueira et al., 2014; Silva et al., 2017), aided by the high capacity for interspecific hybridization and karyotype plasticity (Melo et al., 2001; Melo and Guerra, 2003). Although purple passion fruit are thought to be mostly inbreeding some outcrossing (Bruckner et al., 1995; Brum et al., 2011; de Lira et al., 2016) may have result in morphological variability (Marostega et al., 2017: Ocampo and d’Eeckenbrugge, 2017). Our study confirms the importance of seed descriptors in evaluating genetic variability.

Seed pitting in addition to seed shape was found to be a critical seed descriptor in our study. In the case of the Passifloraceae family’s evolutionary record, seed coats have varied from fovealate (notable seed pitting), to coarsely foveolate, reticulate-foveolate or transversely grooved (Martinez, 2017). Seed lengths varied greatly from very short seeds (1.5 mm) to longer seeds (14 mm); although seed shape tended to be ovoid, obovoid to elliptic. Seed pitting and seed size could have affected the seeds’ relationship with the soil it is planted in and the water available for germination. Seed surface characteristics can be determining factors in water absorption capacity and resulting germination across many plant species (Koomneef et al., 2002). The greater the contact, the more water can be absorbed. Therefore, the structure of the seed coat is an important factor to measure. In the case of the purple passion fruit seed, the exposure area was amplified by level of seed pitting of the fovacous seed coats, increasing the contact surface for water and its retention and absorption. Therefore, another important result of our study was the identification of a direct relationship between germination and seed pitting irrespective of seed size, exhibiting the germination the highest correlation with number of pits (0,789) and area pits (0.854).

We observed that the germination was correlated with the three traits of number, perimeter and area of seed pitting, which indicates that if the seed has a large number of pits over a large area of the seed surface or that the seed pits are large, then the seed germination can be accelerated by several months. Although not part of our study, seed pits reduce the thickness of the seed coat in specific portions of the seed, likely favoring water uptake leading to germination. The variability in the expression of seed pitting among landraces and cultivars is probably related to selection and domestication processes for the species. The increase in fruit size may indirectly select for larger seed size and even if seed pitting remains the same in density the number of seed pits increases.

Water content could also play a role in germination capacity; however, in our study we found no significance in between group (*p*-value = 0.8) or within group (*p*-value = 0.27) variation for hydration based the weight of 100 fresh (PCI) compared to dried seeds (PCIs). Water in seed can be of four types (1) free water in between seed structure; (2) capillary water circulating in seed tissues; (3) cellular water in seed organs; and (4) molecular water bound to other metabolites, molecules or macromolecules of the seed (Craviotto et al., 2009). All of these in conjunction affect seed conservation strategies and seed quality traits of germination rate, viability and vigor. Water content of our seeds varied between 10 to 13% based on seed drying with silica gel. This value was similar to values reported for many crop species as well (soybean for example with previous reference) and in other types of passion fruits. Posada et al. (2012) found that the seed of granadilla, maracuya or yellow passion fruit and gulupa or purple passion fruit (*Passiflora ligularis*, *P. edulis* f. *flavicarpa* and *P. edulis* f. *edulis*, respectively) all had between10 al 12% moisture and that excess drying only affects germination when water content falls to 6%. In future studies we hope to see the effect of seed dessication and moisture content on seed germination and viability in genebanks but for now this was not a priority of our study but rather seed morphology and germination under normal moisture content levels of 10 to 12% were priorities.

In summary, the most important findings of our study were (1) passion fruit seed coats had a rigid structure that was associated with the long dormancy periods; (2) landraces differed from commercial cultivars and genebank accessions in germination time; and (3) there was a positively correlated relationship of germination capacity with the number of seed pits and the area of seed pitting. Seeds with a high number of seed pits over a larger area, had faster germination. Seed structure of purple passion fruit was similar to that of *P. ligularis* based on histological analysis (Cárdenas-Hernández et al., 2011). Three well differentiated layers were likely to be the internal exotegmen, the middle mesotesta, and the external exotestal. Each layer had a different tissue type with hyaline, sclereid and/or palisade cells. In closing, we can see from this study that seed morphology is relevant both today (Crochemore et al., 2003) and in the study of the fossil record of passion fruits (Martinez, 2017). Seed coat structure is critical for survival of the species and their descriptors are often used along with above ground foliar and floral characteristics to characterize genetic diversity. Viability, meanwhile, is important as it can be lost in storage especially in genebank conservation to over-drying of seed or power failure. *In vivo* conservation has its own set of advantages and disadvantages. It is therefore necessary to carry out further morphological and physiological evaluations of the seeds found for landraces and commercial varieties as a complement to the determinations of diversity in passion fruit genotypes held by genebanks. All morphological and genotyping information can be used to find associations with productivity, yield and other morphoagronomic characteristics. These analyses are also useful in the plans for crop improvement or seed certification after varieties have been established in breeding programs.

## Data Availability Statement

All datasets generated for this study are included in the article/Supplementary Material.

## Author Contributions

NC carried out research conceived by LM and MB. All authors wrote sections of the manuscript. MB edited the manuscript. NC and MB prepared figures and tables.

## Conflict of Interest

The authors declare that the research was conducted in the absence of any commercial or financial relationships that could be construed as a potential conflict of interest.

## Footnotes

1 http://www.ipez.es/RWizard
